# Supplementary material for: Electrophysiological responses to conspecific odorants in Xenopus laevis show potential for chemical signaling
Source: PLoS One. 2022 Sep 7;17(9):e0273035. doi: 10.1371/journal.pone.0273035 (PMC9451071; doi:10.1371/journal.pone.0273035)
Supplement: Attachment — Submitted filename: Response to Reviewers.docx [file pone.0273035.s002.docx]

July 31, 2022

Dear Editor,

Thank you and the reviewers for your additional feedback on our manuscript, PONE-D-21-38952. We used this feedback to further revise and improve our manuscript.

Below, we offer a point-by-point response to all new feedback. Reviewer comments are in italics and our responses are in purple text.

We look forward to your response,

Heather J. Rhodes and Melanie Amo

*Reviewer #2: The manuscript has much improved in the revision, in particular the readability for a larger audience. The addition of a schematic figure is welcome.

Minor points:

Figure 1: the opening of the nose (naris) is a white oval in panel A, but a black dot in panel B. Better use the white oval also in panel B, this has the advantage of not having to distinguish between small and large black dots.*

The nares on Fig 1B have been changed to white circles to match Fig 1A.  *Figure 1: The drawing looks like the VNO is accessed from the MC. Can you make the drawing so that VNO looks connected to PC, not to MC?*

The diagram has been altered so that a small bit of the PC (dark blue) sits between the VNO and the MC. This, as well as the description of the anatomy in the paper, should ensure clear understanding of the diagram. *Line 36 „ciliated and microvillous receptor neurons expressing class I OR1, OR2, and V1R receptors“
I think you mean class I and class II ORs, not ' class I OR1, OR2'.*

Our original statement is accurate as written, but we have modified it slightly for clarity; the suggestion by reviewer 2 would be incorrect. Olfactory receptors can be Type 1 (OR1) or Type 2 (OR2). Within the OR1 group, there are two classes: class I and class II. Additionally, there are two types of vomeronasal receptors: V1R and V2R. Adult Xenopus express the class I OR1 (but not class II OR1), OR2, and V1R receptors in the middle cavity. This is reported in several of the cited references, but explained well by Jungblut et al. 2021 on p. 295:

“Early evidence showed that ORs are expressed in both the OE and MCE, but that they belong to different OR classes, with those of the MCE being class I or “fish-like” receptors, and those of the OE class II or “mammalian-like” receptors (Freitag et al. 1995). Both class I and class II receptors belong to the “type 1” or OR1 receptor family, which contains the majority of described OR genes (Niimura and Nei 2005). However, “type 2” or OR2 receptors are also expressed in the adult nose of X. tropicalis; they are found in the MCE, but not the OE (Amano and Gascuel 2012).

*Line 135-136, „a characteristic negative deflection lasting 2-3 seconds with expected timing based on the perfusion and stimulus deliver system described below)“
Unclear. Better say: „a characteristic negative deflection lasting 2-3 seconds, which is the expected timing based on the perfusion and stimulus deliver system described below)“*

Or original statement is accurate as written; the suggestion by reviewer 2 would make the statement inaccurate. The length of the receptor potential is based on the electrophysiological properties of the tissue, not the perfusion system. The perfusion and stimulus deliver system determine the latency, rather than the duration, of the potential. We changed the word “timing” to “latency” to clarify this point.
